# *In vitro* fertilization as an independent risk factor for perinatal complications: Single-center 10 years cohort study

**DOI:** 10.5935/1518-0557.20220041

**Published:** 2023

**Authors:** Neeta Singh, Neena Malhotra, Reeta Mahey, Garima Patel, Monika Saini

**Affiliations:** 1 Department of Obstetrics and Gynaecology, All India Institute of Medical Sciences, New Delhi, India

**Keywords:** ART, IVF, perinatal outcomes

## Abstract

**Objectives:**

Assisted Reproductive Technology (ART) has made great strides in the past forty-years, but no medical treatment comes without side effects. Despite several studies reporting high incidences of perinatal complications, the association is inconclusive. Also, the effect of racially and ethnically distinguished Asian population undergoing ART on perinatal outcomes is not well studied. Therefore, this study attempts to compare various perinatal outcome parameters in ART, and spontaneously conceived singleton pregnancies from a single high-volume tertiary care center.

**Methods:**

This is a retrospective cohort study from a single tertiary infertility center, carried out from January 2011 to September 2020. The study included 1,125 IVF conceived babies (AB group) and 7,193 spontaneous conceived babies (SB group). The groups were compared using the Pearson Chi-square test and adjusted odds ratio, calculated using the multivariate analysis.

**Results:**

Most of the perinatal complications, such as preterm birth (PTB), early preterm birth, low birth weight (LBW), extremely low birth weight, small for gestational age, large for gestational age babies, neonatal intensive care unit (NICU) admission, need for surfactant, meconium aspiration syndrome, neonatal seizures, intraventricular hemorrhage, hypoxic-ischemic encephalopathy, and patent ductus arteriosus was significantly increased in the AB group when compared to the SB group (p<0.05). In-vitro fertilization (IVF) independently increases the risk of LBW (aOR 2.530; 95% CI 2.194-2.917), PTB (aOR 4.004; 95% CI 3.496-4.587), NICU admission (aOR 2.003; 95% CI 1.610-2.492) and neonatal seizures (aOR 9.805; 95% CI 5.755-16.706).

**Conclusions: All** ART-conceived pregnant patients should receive antenatal counselling regarding perinatal complications and should deliver at a tertiary care center with appropriate NICU support.

## INTRODUCTION

Assisted Reproductive Technology (ART) has made great strides in the forty years since the birth of the first *in-vitro* fertilization (IVF) baby in 1978. The field has made incredible advancements and expanded its knowledge, resulting in more than six million babies born worldwide through ART (Kupka *et al*., 2014). IVF has bypassed the hurdles of infertility by conception in a petri dish. This brought forth great enthusiasm for both patients and their clinicians, thus resulting in 2.5 million IVF cycles per year (Fauser, 2019).

Just like a coin has two sides, no medical treatment comes without side effects. There has been caution among clinicians while offering ART because of controversial evidence regarding the increased risk of perinatal complications in IVF conceived babies (Berntsen *et al*., 2019) According to previous studies, either cause of infertility or ovarian stimulation cycle characteristics like the number of oocytes retrieved, type of stimulation cycle or whether it was fresh or frozen embryo transfer or if trophectoderm/cleavage stage biopsy was taken, can bring epigenetic modification in embryos and are thought to be the factor influencing perinatal outcome (Berntsen *et al*., 2019; Chung *et al*., 2006; Pinborg *et al*., 2013). The process of ART manipulates gametes and the embryo, thus affecting epigenetic stability, leading to gamete/embryo origin of adult diseases. Therefore, children born from successful IVF cycles have been reported to have an increased risk of gamete/embryo origin of adult diseases, such as early-onset diabetes, cardiovascular disease, etc. (Romundstad *et al*., 2008; Jiang *et al*., 2017). The neonatal outcomes after IVF have been reported to be worse than in the general population with similar maternal age, parity, and social standing, attributed due to the large proportion of multifetal births after IVF (Koivurova *et al*., 2002). Likewise, a fourfold increase in congenital heart malformations has also been reported in babies born via ART pregnancies (Koivurova *et al*., 2002; Padhee *et al*., 2015).

However, currently available data provides no conclusive evidence regarding IVF as an independent risk factor for these adverse perinatal outcomes. Various maternal confounding factors may predispose them to various perinatal complications; therefore, there is a lack of data regarding IVF as an independent predisposing factor. Further absence of a national ART registry in our country and other developing countries in South Asia, adds to the scarcity of data comparing ART cycles with spontaneously conceived pregnancies in a racially and ethnically distinguished Indian population. This study attempts to investigate IVF as an independent risk factor for various perinatal complications in ART conceived pregnancies to help in counselling and prognosticating the IVF conceived couples.

## MATERIAL AND METHODS

A retrospective study was conducted at an IVF facility of a tertiary care center. It was an observational cohort study involving prospectively collected data from January 2011 to September 2020, which comprised of all infertile females undergoing successful fresh transfer IVF cycles resulting in a singleton pregnancy. For comparison and correlation, a similar cohort of singleton spontaneously conceived females was included as a control. Inclusion criteria involved either primigravida or multigravida with the past history of only one abortion, while patients with co-existing medical disorders (cardiac disease, pregestational diabetes, hypertension, asthma, seizure disorder, hypothyroidism), *in-vitro* fertilization with donor oocyte cycle or frozen embryo transfer were excluded from the study. 10,360 females fulfilled these criteria; however, due to incomplete medical records only 8,318 were included in the study. Among these, 7,193 conceived spontaneously, while the remaining 1,125 conceived via IVF. All patients received standard antenatal care as per the departmental protocol and delivered at least one live birth after the viable age i.e., 26 weeks. Both the ART and control group were analyzed for perinatal complications.

Perinatal outcome measures included: gestation at delivery, the newborn weight, Apgar scores, mode of delivery, the requirement of NICU admission, oxygen requirement (if any), need for resuscitation/ventilatory support/surfactant administration, any congenital malformation, and neonatal mortality.

Low birth weight was defined as baby weight at birth less than 2500 gr (WHO, 2004) and preterm birth, as defined as delivery before 37 weeks of gestation (WHO, 2018). Low birth weight is further categorized into very low birth weight (VLBW, <1500 g) and extremely low birth weight (ELBW, <1000g) (WHO, 2004). Similarly preterm birth is further classified into early (<34 weeks) and late preterm birth (34-37 weeks) for better evaluation (Chawanpaiboon et al., 2019). Neonatal death has been defined by the World Health Organization (WHO) as “deaths among live births during the first 28 completed days of life” (WHO, 2006).

### Statistical analysis

The data was compiled on an excel spreadsheet and analyzed using SPSS.v.23 software. The data were divided into two groups: (a) Babies conceived via ART (AB) and (b) Spontaneously conceived babies (SB). Variables amongst the groups were compared using the Pearson Chi square test and a *p-*value <0.05 was considered statistically significant. Multivariate analysis was performed; adjusted odds ratios (aORs), 95% confidence intervals (CIs), and 2-sided *p* values were calculated. Differences were considered statistically significant if the effect estimate excluded 1.0 from the 95% CI and the 2-sided *p*-value was <0.05.

## RESULTS

Out of the 8,318 females included in the study, 1,125 females conceived through ART. The mean age of ART conceived females was higher than spontaneously conceived females (31 years *versus* 26 years), which was statistically significant (*p*<0.001). The distribution of maternal age in the two groups is shown in Table 1. The majority (>50%) of the AB group females were more than 30 years of age, contrary to the SB group females who were mostly confided in the 21-30 years age group.

**Table 1:** Maternal age distribution in the two groups.

Maternal age	AB Group (ART conception) N=1125 (%)	SB Group (Spontaneous conception) N=7193 (%)	*p*-value
15-20 years	5 (0.4%)	308 (4.3%)	<0.001
21-25 years	109 (9.7%)	2827 (39.3%)	<0.001
26-30 years	391 (34.8%)	3029 (42.1%)	<0.001
31-35 years	388 (34.5%)	841 (11.7%)	<0.001
36-40 years	168 (14.9%)	167 (2.3%)	<0.001
>40 years	64 (5.7%)	21 (0.3%)	<0.001

### Perinatal complications

The majority of the perinatal complications like preterm birth (PTB), early preterm birth, low birth weight (LBW), extremely low birth weight, small for gestational age (SGA), large for gestational age (LGA) babies, neonatal intensive care unit (NICU) admission, need for surfactant, meconium aspiration syndrome (MAS), neonatal seizures, intraventricular hemorrhage (IVH), hypoxic-ischemic encephalopathy (HIE) and patent ductus arteriosus (PDA) was significantly increased in AB group as compared to the SB group (*p*<0.05). Whereas the incidence of transient tachypnoea of new-born (TTN), hyaline membrane disease (HMD), congenital malformations and neonatal death was similar in the two groups (Table 2).

**Table 2:** Perinatal complications in IVF singletons and spontaneously conceived control population.

Perinatal outcome	AB Group (ART conception) N=1125 (%)	SB Group (Spontaneous conception) N=7193 (%)	*p*-value
Mean gestation at delivery (weeks)	36±3	38±2.3	<0.001
Mean weight of new-born (gr)	2484±724	2784±578	<0.001
Low birth babies (<2500 gr)	572 (50.8%)	2092 (29.1%)	<0.001
Extreme low birth weight (<1000 gr)	24 (2.1%)	45 (0.6%)	<0.001
Early preterm birth (<34 weeks)	202 (18%)	412 (5.7%)	<0.001
Preterm birth (34-37 weeks)	558 (49.6%)	968 (13.5%)	<0.001
Small for gestational age (SGA) babies	78 (7.2%)	448 (6.8%)	0.05
LGA babies	137 (12.6%)	675 (10.3%)	0.05
Chorioamnionitis	32 (2.8%)	3 (0.01%)	<0.001
NICU admission	172 (23.6%)	595 (8.3%)	<0.001
1 min Apgar <7	113 (10.1%)	487 (6.8%)	<0.001
5 min Apgar <7	43 (3.9%)	130 (1.8%)	<0.001
Supplemental oxygen	41 (3.6%)	519 (7.2%)	<0.001
Transient tachypnoea of new-born (TTN)	75 (6.7%)	578 (8%)	0.112
Meconium aspiration syndrome (MAS)	31 (2.8%)	59 (0.8%)	<0.001
Hyaline membrane disease (HMD)	14 (1.2%)	126 (1.8%)	0.219
Intubation	168 (15%)	219 (3%)	<0.001
Surfactant use	53 (4.7%)	89 (1.2%)	<0.001
Neonatal Seizures	73 (6.5%)	33 (0.5%)	<0.001
Intraventricular Hemorrhage (IVH)	23 (2%)	50 (0.7%)	<0.001
Hypoxic-Ischemic Encephalopathy (HIE)	61 (5.4%)	61 (0.8%)	<0.001
Congenital malformations	21 (2.9%)	185 (2.6%)	0.626
Patent ductus arteriosus (PDA)	37 (3.3%)	33 (0.5%)	<0.001
Neonatal death	29 (2.6%)	130 (1.8%)	0.079

### IVF as an independent risk factor for LBW and PTB

Babies conceived with ART delivered at an earlier gestation (36±3 weeks *vs*. 38±2.3 weeks, *p*<0.001) and had statistically significantly lower birth weight (2484±724gr *vs*. 2784±578gr, *p*<0.001) compared to spontaneously conceived babies. There are various independent risk factors of LBW and PTB like pre-eclampsia, gestational hypertension, maternal age, and antepartum hemorrhage. IVF also independently increases the risk of LBW and PTB by 2.5 times (*p*<0.001) and 4 times (*p*<0.001), respectively (Tables 3 and 4).

**Table 3:** Multivariate analysis of risk factors for low birth weight in the present study.

Variable	Multivariate analysis			*p*-value
	OR	95% CI		
Pre-eclampsia	5.012	3.463	7.255	<0.001
Gestational hypertension	1.814	1.532	2.149	<0.001
Maternal age	1.019	1.007	1.030	0.001
Chorioamnionitis	6.076	2.485	14.858	<0.001
Antepartum hemorrhage	4.279	2.316	7.906	<0.001
Placenta previa	1.913	1.070	3.418	.029
Mode of conception (IVF *vs*. SC)	2.530	2.194	2.917	<0.001

**Table 4:** Multivariate analysis of risk factors for preterm birth in the present study.

Variable	Multivariate analysis			*p*-value
	OR	95% CI		
Pre-eclampsia	4.863	3.415	6.925	<0.001
Gestational hypertension	2.050	1.715	2.450	<0.001
Polyhydramnios	2.592	1.394	4.820	.003
Chorioamnionitis	47.272	6.385	349.968	<0.001
Antepartum hemorrhage	9.847	5.002	19.384	<0.001
Placenta previa	3.082	1.682	5.647	<0.001
Oligohydramnios	2.649	2.027	3.462	<0.001
Mode of conception (IVF *vs*. SC)	4.004	3.496	4.587	<0.001

### IVF as an independent risk factor for small for gestational age (SGA)

Babies of the AB group were more prone to be diagnosed with SGA (7.2% *vs*. 6.8%, *p*=0.05) than in the SB group. But on eliminating the covariates like APH, maternal age, and placenta previa, IVF has slightly increased risk for SGA, but not statistically significant (*p*=0.058) (Table 5).

**Table 5:** Multivariate analysis of risk factors for small for gestational age in the present study.

Variable	Multivariate analysis			*p*-value
	OR	95% CI		
Antepartum hemorrhage	2.598	1.083	6.233	0.032
Placenta previa	0.000	0.000	0.000	0.997
Maternal age	0.969	0.949	0.989	0.003
Mode of conception (IVF *vs*. SC)	1.298	.992	1.700	0.058

### IVF as an independent risk factor for NICU admission

There is a higher incidence of NICU admission for the babies from the AB group compared to those from the SB group (23.6% *vs*. 8.3%, *p*<0.001). Contributing factors like PTB, LBW, SGA, congenital anomalies, and PDA independently increase the risk of NICU admission. IVF itself is an independent predictor of NICU admission, and it doubles the risk as compared to the SB group (Table 6).

**Table 6:** Multivariate analysis of risk factors for NICU admission in the present study.

Variable	Multivariate analysis			*p*-value
	OR	95% CI		
Preterm birth	3.815	3.114	4.673	<0.001
Low birth weight	2.105	1.695	2.615	<0.001
Small for gestational age	2.399	1.893	3.040	<0.001
Congenital malformations	6.167	4.401	8.641	<0.001
Patent ductus arteriosus	2.890	1.553	5.379	0.001
Meconium aspiration	1.415	.786	2.548	0.247
Low Apgar score	1.872	1.376	2.548	<0.001
Mode of conception (IVF *vs*. SC)	2.003	1.610	2.492	<0.001

### IVF as an independent risk factor for Neonatal seizures

There is a relatively higher incidence of neonatal seizures for the babies from the AB group compared to those in the SB group. Contributing factors, such as PTB, LBW, extremely LBW or very preterm delivery, risk of IVH or HIE. IVF itself is an independent predictor of neonatal seizures (Table 7).

**Table 7:** Multivariate analysis of risk factors for Neonatal seizures in the present study.

Variable	Multivariate analysis			*p*-value
	OR	95% CI		
Antepartum hemorrhage	4.098	1.213	13.848	.023
Chorioamnionitis	.525	.139	1.975	.340
Oligohydramnios	.840	.197	3.583	.814
Low birth weight	1.235	.672	2.270	.497
Low Apgar score	.795	.370	1.709	.557
Cardiac malformations	1.594	.393	6.471	.514
Preterm birth	1.522	.820	2.828	.183
Hypoxic-ischemic encephalopathy (HIE)	26.632	14.919	47.540	<0.001
Intraventricular hemorrhage (IVH)	8.039	3.269	19.768	<0.001
Mode of conception (IVF *vs*. SC)	9.805	5.755	16.706	<0.001

## DISCUSSION

The innovations of the 21^st^ century in the field of ART improved pregnancy outcomes. But the positive outcomes always come at the expense of some adverse events. Recent data associated various perinatal complications with IVF conception and caused apprehension among patients and their clinicians. Evidence from previous studies was confounded, and there was no direct correlation/association of IVF with perinatal complications till now.

Among the IVF conceived babies in the current cohort, 67.6% were PTB and 50% were LBW, compared to the spontaneously conceived babies. Similarly in a metanalysis by Pandey *et al*. (2012), these high rates of PTB and LBW can be accounted for, due to an increased rate of induction of labor due to raised incidences of PIH, GDM, APH and other pregnancy induced co-morbidities in IVF conceived pregnancies. Also, an increased frequency of spontaneous PTB before 34 weeks was observed in IVF conceived pregnancies. Subsequently, this led to an increase in detection of patent ductus arteriosus (PDA), in concordance with the results observed in other studies too (Connuck *et al*., 2002). Though the incidence of LBW and VLBW is decreasing as evident in a study of the perinatal outcome of 62,379 ART singletons born between 1988 and 2007 in four Nordic countries from the CoNARTaS group (Henningsen *et al*., 2011), there were significant adjusted risk between 1.41 and 2.04 for PTB and 1.6-1.7 for LBW in pregnancies conceived through IVF/ICSI (Marino *et al*., 2014). Although 50.8% of the babies were less than 2500gr; only 7.2% were small for gestational age (SGA), highlighting PTB as the primary cause for LBW. PIH being the primary contributing factor for SGA babies, causes placental hypoperfusion; thereby preventing fetal growth (Friedman *et al*., 1991). The current study also observed an increased incidence of SGA in the AB group, as compared to the SB group, but ART is not an independent risk factor for such babies. This might be contrary to some published literature (Pandey *et al*., 2012), but the recent study by Glatthorn *et al*. (2021), in tandem with our study, concluded for the reduced risk of SGA births reflecting changes in modern infertility treatments, maternal lifestyle, and better compliance to prenatal care.

Current data support an increased risk of low birth weight babies associated with controlled ovarian stimulation and manipulation of gametes in fresh transfer cycles, because it affects endometrial receptivity, implantation, and early pregnancy (Shih *et al*., 2008)distribution of birthweight, z-score, LBW (<2500 g. Conflicting to this existent literature (Korosec *et al*., 2016), our study population also demonstrated a positive correlation between LGA babies and fresh IVF cycles. This might be due to a higher incidence of GDM in the study population (due to Asian ethnicity), leading to excess weight gain for both mother and the fetus (Chen *et al*., 2019). Therefore, the correlation of IVF pregnancies with the perinatal outcomes should be compared in different study populations, according to race and ethnicity as they will be predisposed to different maternal complications - which can adversely affect the neonatal outcomes.

Meconium-stained liquor is a sign of fetal compromise, which leads to poor neonatal outcomes. Placental insufficiency, maternal hypertension, pre-eclampsia, and oligohydramnios induce hypoxic stress and result in anal sphincter relaxation, leading to the intrauterine passage of meconium (Mohammad et al., 2018). Meconium aspiration syndrome (MAS) occurs in 4-10% of the neonates exposed to meconium stained liquor in the milieu intrapartum (Raju et al., 2010). Because the IVF mothers frequently develop multiple pregnancy induced co-morbidities, their babies are most prone to MAS as witnessed in this study too.

Although around half of the babies born via IVF conception were late preterm; both early and late preterm births were prone to NICU admission. Also, these babies had poor Apgar scores at 1 and 5 minute-intervals, making them predisposed to oxygen supplementation and intubation. Though its exact cause remains elusive, high rates of caesarean sections and low weight at birth are the primary contributors. The rate of respiratory distress syndrome (RDS) and transient tachypnoea of new-born was similar between the two groups, as per observed by Ahmad *et al*. (2019). This phenomenon is chiefly attributed to our vigilant and strict antenatal and fetal surveillance, involving all IVF conceived pregnancies with early prophylactic corticosteroid administration for fetal lung maturity to patients predisposed to preterm delivery or requiring early induction of labor/delivery due to antenatal medical comorbidities.

In tandem with our study, IVF has been considered an independent risk factor for intraventricular hemorrhage (IVH) in neonates (Shariat *et al*., 2019). Various maternal factors associated with IVF as well as an increased predisposition to IVH include chorioamnionitis, infection, inflammation, antenatal hemorrhage, prematurity, and low birth weight (Luque *et al*., 2014; Shankaran *et al*., 2014). These risk factors also increase predilection for the risk of hypoxic ischemic encephalopathy (HIE) (Parker *et al*., 2018). Although the literature supporting IVF as a causative agent for HIE is scarce, our study provides robust evidence demonstrating an association between the two. HIE is also among the most common causes of symptomatic neonatal seizures; whereas IVH predisposes to neonatal seizures only when associated with parenchymal brain injury (Glass *et al*., 2016)burden, and short-term outcomes of seizures in neonates monitored with continuous video-electroencephalogram (cEEG. While none of the currently published literature provides evidence regarding an association between neonatal seizures and ART conception (Wang *et al*., 2017), our study reports a 12 fold increased risk of neonatal seizures in IVF babies. This observation can be accounted for due to very or extremely preterm birth, or very low birth weight or incomplete corticosteroid cover predisposing these babies to IVH and subsequently neonatal seizures. IVF-conceived mothers are prone to develop GDM, leading to birth of the LGA babies, which can lead to hypoglycemia and hypomagnesemia in neonates, again predisposing them to neonatal seizures.

Though a higher rate of birth defects has been reported in children conceived through IVF (Pandey et al.,2012; Hansen et al.,2013), and this risk further elevated when comparison was made with respect to major birth defects (Hansen et al., 2013), but only 2.9% of the babies in the AB group of our study had congenital malformations; such as cardiac anomalies, neural tube defects, cleft lip/palate, gastrointestinal malformations, and genitourinary malformations. This incidence was comparable to spontaneously conceived babies and also the recent literature lacks any conclusive evidence to support increased congenital anomalies in ART babies (Hoorsan et al., 2017; Banker et al., 2019). Also, the previous evidence was confounded by the inclusion of multiple order pregnancies, but the most recent population-based cohort study associated ART singletons with an increased risk of major non-chromosomal birth defects (Luke et al., 2021).

The previous literature reported an increased risk of perinatal mortality in ART conceived babies, but there is marked heterogeneity since it includes stillbirths and neonatal deaths. Similarly, singletons conceived through ART had an increased risk of infant mortality from birth up to 1 year of life, predominantly in the early neonatal period (Rodriguez-Wallberg *et al*., 2020); whereas our study demonstrated a similar rate of neonatal death in the AB group as compared to the SB group. This might be due to strict and vigilant fetal surveillance and the availability of one of the best NICU facilities at our center.

This study assessed IVF-conception outcomes from a high volume tertiary care center in a country that has the second largest population and houses more than a million IVF, facilities providing infertility services at a much lower cost compared to the western world (Bansode, 2017). The comparative series describes the largest cohort studied amongst current developing nations. Lack of an established national registry limited our sample size, restricting us from achieving a larger cohort as studied in western countries. This study highlighted various associations between IVF and perinatal complications. This would provide a benchmark to create awareness among fertility specialists, especially from developing nations regarding various possible complications related to IVF and would wary them to be more vigilant in diagnosing as well as treating it. IVF-pregnancy is usually deemed high risky and requires vigilant monitoring and follow-up subsequent to its effect on fetal outcomes.

## CONCLUSION

ART births are strongly associated with poor perinatal outcomes. Thus, all infertile patients scheduled for high technology infertility treatments should be informed and prognosticated regarding increased perinatal risk in case of a successful IVF cycle. Careful pre-conceptional counselling and delivering at a tertiary care center with appropriate NICU support are keystones to optimize perinatal outcomes.
